# Epithelial stratification and placode invagination are separable functions in early morphogenesis of the molar tooth

**DOI:** 10.1242/dev.130187

**Published:** 2016-02-15

**Authors:** Jingjing Li, Lemonia Chatzeli, Eleni Panousopoulou, Abigail S. Tucker, Jeremy B. A. Green

**Affiliations:** Department of Craniofacial Development & Stem Cell Biology, King's College London, London SE1 9RT, UK

**Keywords:** Ectodermal organ, Morphogenesis, Placode, Invagination, Asymmetric cell division

## Abstract

Ectodermal organs, which include teeth, hair follicles, mammary ducts, and glands such as sweat, mucous and sebaceous glands, are initiated in development as placodes, which are epithelial thickenings that invaginate and bud into the underlying mesenchyme. These placodes are stratified into a basal and several suprabasal layers of cells. The mechanisms driving stratification and invagination are poorly understood. Using the mouse molar tooth as a model for ectodermal organ morphogenesis, we show here that vertical, stratifying cell divisions are enriched in the forming placode and that stratification is cell division dependent. Using inhibitor and gain-of-function experiments, we show that FGF signalling is necessary and sufficient for stratification but not invagination as such. We show that, instead, Shh signalling is necessary for, and promotes, invagination once suprabasal tissue is generated. Shh-dependent suprabasal cell shape suggests convergent migration and intercalation, potentially accounting for post-stratification placode invagination to bud stage. We present a model in which FGF generates suprabasal tissue by asymmetric cell division, while Shh triggers cell rearrangement in this tissue to drive invagination all the way to bud formation.

## Abstract (precis)

**Summary:** During tooth development in mice, FGF-dependent vertical cell divisions thicken the tooth placode while Shh drives cell rearrangements that cause invagination.

## INTRODUCTION

Expansion, thickening, stratification and bending of an epithelium are morphogenetic modules that are iteratively used to determine the size and shape of an organ (St Johnston and Sanson, 2011). These modules can be further resolved into individual cell behaviours, including proliferation, shape change, rearrangement and migration. Combinations of these elements create diverse modes of epithelial reorganisation in organogenesis (Montell, 2008). One mode that occurs at the initiation of many organs is the formation of a placode (local thickening), which then invaginates to form an organ bud. In cranial sensory organs such as the optic, otic and olfactory primordia, epithelium thickens by columnarisation of a single-cell layer and invaginates to form a hollow vesicle (Park and Saint-Jeannet, 2010). In non-sensory ectodermal organs, including the teeth, mammary glands and hair follicles, the placode grows into a solid epithelial peg or bud that invades the underlying mesenchyme (Biggs and Mikkola, 2014). Thickening (initially by cell columnarisation), stratification (the formation of cell layers) and bending (invagination) of the epithelium are shared by non-sensory ectodermal organs at the initiation of their development before they diverge into their mature forms (Pispa and Thesleff, 2003).

Signal networks that regulate the early development of ectodermal organs have been extensively investigated, with much focus on the reciprocal interactions between epithelium and mesenchyme (Jernvall and Thesleff, 2000; Mikkola and Millar, 2006; Sennett and Rendl, 2012). Fibroblast growth factor (FGF), Wnt, transforming growth factor β (TGFβ) superfamily members including activin and BMP, hedgehog (Hh) and, more recently, ectodysplasin (Eda) signalling, have been implicated (Biggs and Mikkola, 2014). For example, in tooth development, an early expression of Shh marks the odontogenic band, which is the competent region for tooth formation on the maxillary and mandibular processes (Keränen et al., 1999). Shh expression becomes restricted to the regions where individual incisors and molars form (Cobourne et al., 2001; Keränen et al., 1999), and pharmacological inhibition of Shh signalling arrested tooth development at the initiation stage (Cobourne et al., 2001). Epithelial (keratin 14 promoter-driven) conditional knockout of Shh signalling, which takes effect from the middle of the placodal stage, resulted in misshapen teeth (Dassule et al., 2000). Members of the FGF family are expressed in a wide range of developing ectodermal organs, including the teeth, salivary glands, mammary glands and hair follicles, and perturbation of the FGF pathway arrests tooth and salivary gland development prior to the bud stage, reducing the number of hair follicles and impairing placode formation in the mammary glands (Hosokawa et al., 2009; Li et al., 2014; Mailleux et al., 2002; Ohuchi et al., 2000; Petiot et al., 2003).

Compared with the genetic regulation of the early development of ectodermal organs, cellular mechanisms that form placodes and buds are poorly described. A common view is that the elevated proliferation of placode cells generates local stratification and invagination (Linde, 1984). Although early studies of mitotic index in placodes revealed similar or lower levels compared with the surrounding epithelium in tooth, mammary and hair placodes (Balinsky, 1950; Osman and Ruch, 1975; Wessells and Roessner, 1965), more recently evidence of proliferation bursts was shown in hair placodes (Magerl et al., 2001; Schmidt-Ullrich et al., 2006). Another suggestion is that vertically orientated cell divisions cause stratification and invagination (Linde, 1984). Such perpendicular divisions, which generate an apical and a basal daughter, drive stratification in a number of tissues (reviewed by Panousopoulou and Green, 2014), including mouse embryonic interfollicular (i.e. non-placodal) epidermis (Lechler and Fuchs, 2005) and *Xenopus* neurectoderm (Tabler et al., 2010) but, although cited as a textbook fact (Nanci and Ten Cate, 2013), the theory that the tooth placodes form by orientated cell division has never been tested experimentally. A third cellular mechanism for stratification is simple delamination, in which cells detach from the basement membrane independently of cell division and migrate to the suprabasal space (Williams et al., 2014). Although both orientated cell division and simple delamination have been characterised in the development of epidermis and neuroepithelium (Wodarz and Huttner, 2003), it is currently unknown in the early development of ectodermal organs which, if either, of these is responsible for creating the placode (Kulukian and Fuchs, 2013). Studies in mammary gland and epidermis have implicated a fourth process: centripetal cell convergence (Ahtiainen et al., 2014; Propper, 1978). However, whether cells converge within, under or over the pre-existing epithelial layer has not been established, and the relationship of placode thickening to placode invagination is not clear.

In this study, we used early development of the mouse molar to investigate cell dynamics and their relationship to signalling in placode formation and invagination. We found that perpendicular divisions, although initially restricted to prospective placodes, rapidly become more widespread. We further found using inhibitors that cell proliferation is absolutely required for placodal stratification, but not for invagination or bud formation once stratification has begun. Remarkably, stratification and invagination could be separated according to signalling pathway: FGF signalling is necessary and sufficient for proliferation and stratification, whereas Shh is required for convergence, invagination and bud neck formation. Together, these resolve ectodermal placode formation and invagination into two simple morphogenetic elements.

## RESULTS

### Spindle orientation in early tooth placode stratification and invagination

**Fig. 1.**
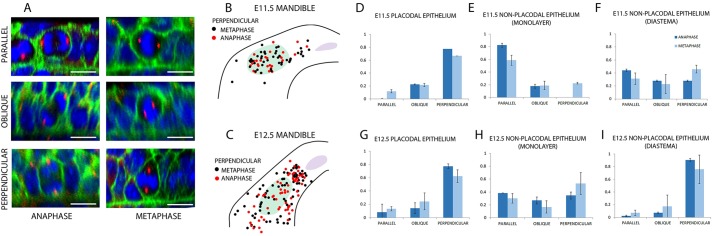
**Spindle orientation in stratifying and invaginating dental epithelium.** (A) Examples of anaphase and metaphase cells showing different orientations in a dental epithelium. Blue, DAPI; green, β-catenin; red, γ-tubulin. Scale bars: 10 μm. (B,C) Perpendicular divisions mapped on mandible at E11.5 (B) or E12.5 (C). Black dots, metaphase cells; red dots, anaphase cells; light green, molar region; lilac, incisor region. (D-F) Distributions of spindle orientation in E11.5 tooth placode (D), non-placodal monolayer (E) and non-placodal diastema (F). (G-I) Distributions of spindle orientation in E12.5 tooth placode (G), non-placodal monolayer (H) and non-placodal diastema (I). *n*=9 specimens (three from each of three litters). Error bars indicate s.d.

To assess mitotic spindle orientation in initiating tooth placode and adjacent non-placode epithelium, we stained whole mandibles of E11.5 and E12.5 mouse embryos for γ-tubulin, β-catenin and with DAPI to show, respectively, centrosomes, cell boundaries and nuclei. Since we were concerned primarily with cells leaving the basal layer (i.e. the layer of cells touching the basal lamina), we analysed spindle orientations relative to the basal lamina in this layer only (Fig. 1A). At E11.5, when a placode is just distinguishable from the surrounding oral epithelium as a thickened but hardly invaginated epithelium, perpendicular divisions were mostly in the placodal region (Fig. 1B) but, by E12.5, the distribution had expanded proximally and distally to include the diastema, which is the region of epithelium between the incisor and the molar thickenings (Fig. 1C) (Yuan et al., 2008), which at this stage is noticeably thinner than the placodes. Quantifying perpendicular divisions as a proportion of total divisions showed that, at E11.5, spindles are predominantly perpendicular within the placode (Fig. 1D), randomly orientated in the prospective diastema (Fig. 1F) and predominantly parallel in other non-placodal epithelium (Fig. 1E). By E12.5, when the epithelium is actively invaginating to form a tooth bud, spindles were now perpendicular not only in the placode (Fig. 1G) but also in the diastema (Fig. 1I), remaining random elsewhere (Fig. 1H). Transient buds are known to appear in this region at this stage (Prochazka et al., 2010). Although mitotic spindles rotate during metaphase in some systems (e.g. da Silva and Vincent, 2007), metaphase and anaphase spindle orientations were similar throughout (Fig. 1D-I). Together, these data suggested that because perpendicular divisions (i.e. with vertical spindles) showed strong spatial correlation with thickening epithelia, they could contribute to tooth placodal stratification.

### Cell division is required for stratification but not invagination of the dental epithelium

If perpendicular cell divisions contribute to stratification, a strong prediction is that stratification is cell division-dependent. To test this, we cultured frontal slices of E11.5 and E12.5 mouse maxilla and mandible containing tooth placodes with the well-established proliferation inhibitor cocktail of hydroxyurea and aphidicolin (HU+APH) (Harris and Hartenstein, 1991), using vehicle (DMSO)-treated contralateral placodes from the same slice as controls. Proliferation inhibition was confirmed by observing reduced BrdU incorporation into the tissue (Fig. S1A).

**Fig. 2.**
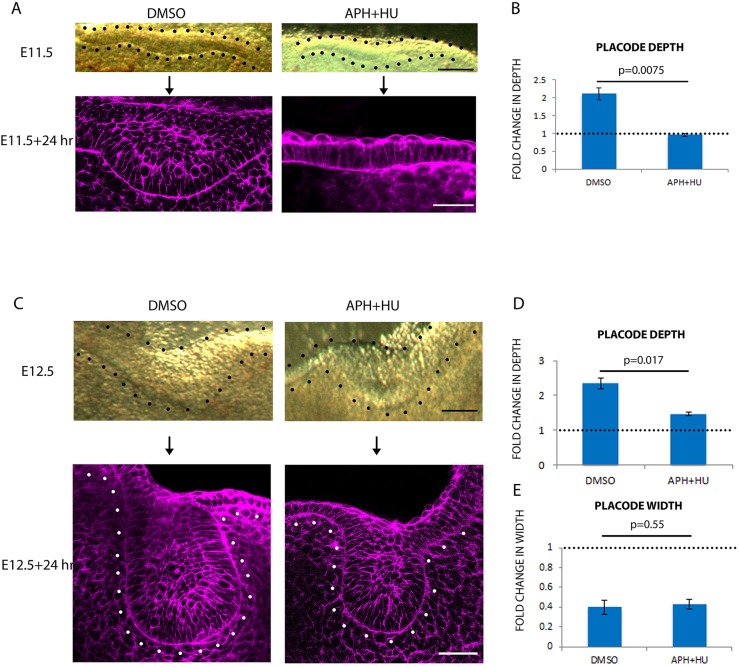
**Cell proliferation is required for epithelial stratification but not for invagination.** (A) Frontal slices of E11.5 tooth placodes cultured for 24 h with vehicle (DMSO) or proliferation inhibitors (APH+HU). (Top row) Brightfield images showing the initial morphology. (Bottom row) Fixed slices stained with phalloidin (magenta). (B) Quantification of placode depth in A. *n*=3. (C) Frontal slices of E12.5 tooth placodes cultured for 24 h with vehicle or proliferation inhibitors. (D,E) Quantification of placode depth (D) and width (E) in C. Black and white dots outline the epithelium. *n*=9 specimens (three from each of three litters). Error bars indicate s.d. Scale bars: 50 μm.

When cultured from E11.5 for 24 h, control placodes formed stratified epithelium and underwent invagination typical for a placode at E12.5 (Fig. 2A). By contrast, placodes treated with HU+APH failed to stratify into multiple layers or invaginate and retained an E11.5 morphology after culture (Fig. 2A). Quantification of the change in depth showed that control placodes deepened 2-fold over 24 h, whereas with proliferation inhibitors the tooth placodes failed to thicken at all (Fig. 2B).

When cultured from E12.5, at which stage the tooth placodes were already stratified and beginning to invaginate into the mesenchyme, both control and proliferation-inhibited placodes grew into bud shapes, resembling the E13.5 molar morphology with a narrowed ‘neck’ near the epithelial surface of the buds (Fig. 2C). Although placodes treated with HU+APH were significantly shallower than the controls (Fig. 2D) they showed a reduced but still significant fold change in depth of almost 2 over the 24 h of culture (Fig. 2D). Likewise, both inhibitor-treated and untreated placodes narrowed substantially in the culture period (Fig. 2E; fold change in width less than 1) with no significant difference between DMSO and APH+HU treatments. These results indicated that, from E12.5, morphogenesis to form a bud is not dependent on cell proliferation.

### Proliferation controls the generation of suprabasal cells and the size of tooth placode

**Fig. 3.**
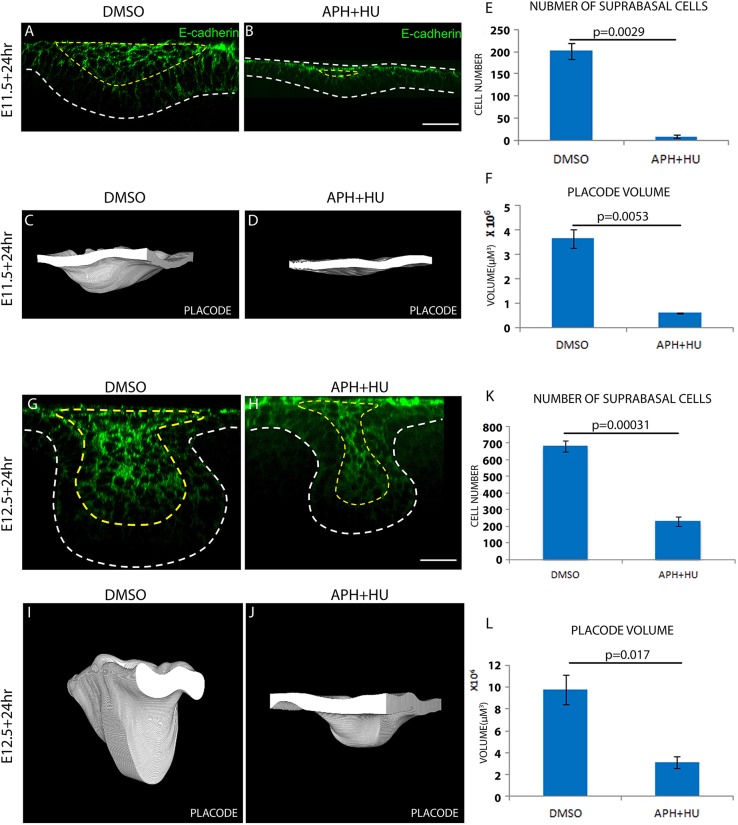
**Cell proliferation controls suprabasal cell formation and the size of the early tooth.** (A,B,G,H) E-cadherin (DECMA-1) immunofluorescence showing suprabasal cells of tooth placodes cultured from E11.5 (A,B) or E12.5 (G,H) with vehicle (DMSO) or proliferation inhibitors (APH+HU) for 24 h. (C,D,I,J) 3D rendering of tooth placodes cultured from E11.5 (C,D) or E12.5 (I,J) for 24 h with vehicle or proliferation inhibitors. White areas show the front-facing (virtually) cut face of the epithelium. (E) Quantification of suprabasal cell number in A and B. (F) Measurements of placode volume in C and D. (K) Quantification of suprabasal cell number in G and H. (L) Measurements of placode volume in I and J. For all quantifications, *n*=9 specimens (three from each of three litters). Error bars indicate s.d. Scale bars: 50 μm.

The slice culture results above suggested that when proliferation was inhibited, it was the growth in volume but not the ability of the placode to shape itself and invaginate that was impaired. As suprabasal cells are the cell population that makes up the invaginated volume of the placode, we next examined whether the number of suprabasal cells was affected by the inhibition of proliferation. To do this, instead of culturing frontal slices, which only include part of the placode in each slice, we cultured whole mandibular explants from E11.5 and E12.5 embryos to examine the impact on entire placodes. After culturing for 24 h, mandibular explants were fixed and stained for E-cadherin, a known marker enriched in suprabasal cells (Chiu et al., 1992) (Fig. 3A,B,G,H). The number of E-cadherin-positive cells was reduced to almost zero when proliferation was inhibited from E11.5 for 24 h (Fig. 3E). Similarly, cultures inhibited from E12.5 had essentially the same number of suprabasal cells as control placodes inhibited from E11.5 for 24 h (Fig. S2A), suggesting that inhibition of proliferation had arrested any increase in suprabasal cell number substantially or even completely. A similar reduction in basal cell number was observed (Fig. S2B,C). These effects on cell number were similarly reflected in tooth placode volume as assessed by 3D rendering of treated and control placodes/buds (Fig. 3C,D,F,I,J,L) and suprabasal tissue only (Fig. S2D-G). F-actin (phalloidin staining, Fig. 2), β-catenin (Fig. S1A), nuclear morphology (DAPI staining, data not shown) and *Shh* gene expression (Fig. S1B) were not affected by the cell proliferation inhibitors, ruling out substantial cell death or general loss of cell viability and specific gene expression.

Together, these results validated our slice culture findings and we concluded that proliferation is required for the generation of suprabasal cells and, consequently, the growth in volume of the tooth placode, but not its invagination after E12.5.

### FGF signalling is both necessary and sufficient for oral epithelial stratification

The finding that proliferation is only required for stratification but not invagination revealed that these are two distinct processes that can be uncoupled and, as such, might be regulated by distinct molecular pathways. FGF signalling has been found to be multifunctional, participating in almost all stages of tooth development (Li et al., 2014), and several mutants of FGF ligands and receptors exhibit arrest of tooth development at early stages (Hosokawa et al., 2009; Kettunen et al., 2007; Neubüser et al., 1997; Ohuchi et al., 2000). However, phenotypes of mutants in single FGF and FGF receptor genes are complicated by the large number of FGF family member and receptor combinations and their potentially compensatory roles. Moreover, although FGF mutants were reported to have reduced proliferation in the tooth, it was unclear whether proliferation loss was the primary cause of the observed dysmorphology or incidental to it. It was also unclear, because of the relatively limited temporal control provided by genetic knockout, when FGF signalling is required for tooth development. We therefore reinvestigated the function of the FGF pathway using a pan-FGF receptor inhibitor SU5402 (SU), which we found effective in our explant culture system (Fig. S3A) and which also provided an advantage in allowing precise temporal control of perturbation of the signalling pathway.

**Fig. 4.**
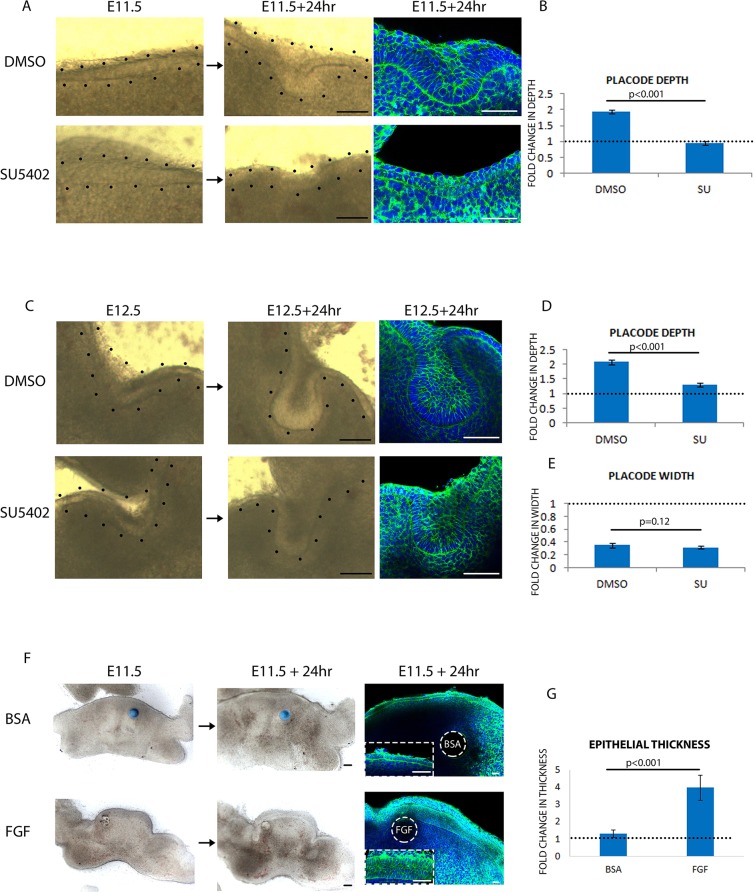
**FGF is both necessary and sufficient for epithelial stratification.** (A,C) Frontal slices of tooth placode cultured from E11.5 (A) or E12.5 (C) with vehicle (DMSO) or FGF inhibitor (SU5402) for 24 h. Left and middle columns are brightfield images showing the starting and ending morphology of the placodes; the right-hand column shows placodes fixed after culture and stained with DAPI (blue) and phalloidin (green). Black dots outline tooth placodes. (B) Quantification of placode depth in A. (D,E) Quantification of placode depth (D) and width (E) in C. (F) Induction of epithelial stratification on E11.5 mandible frontal slices with BSA-soaked or FGF-soaked beads. Insets are magnifications of the tongue epithelium. (G) Quantification of epithelial stratification in F. For all quantifications, *n*=9 specimens (three from each of three litters). Error bars indicate s.d. Scale bars: 50 μm, except 25 μm in insets in F.

Upon culturing frontal slices of the tooth placode from E11.5 for 24 h with either DMSO or SU, the DMSO control placodes developed into an E12.5 morphology, whereas SU treatment caused a failure in stratification in the placodal area (Fig. 4A) and a significant reduction in the deepening of the placode (Fig. 4B). Placodes treated with SU from E12.5 for 24 h invaginated and formed a tooth bud (Fig. 4C) but fold change in depth was significantly reduced, although the value was still greater than 1, indicating some degree of downward invagination (Fig. 4D). However, SU treatment had almost no effect on the decrease in placode width (Fig. 4E). Placode volume measurements confirmed that SU placodes were significantly smaller than those of controls (Fig. S3D-F). Together, these experiments show that FGF signalling is required for epithelial stratification and the generation of suprabasal cells, but is not required for placode invagination as such after stratification has taken place. In other words, the effects of inhibiting FGF signalling are virtually identical to those of proliferation inhibition shown above.

To test whether FGF is sufficient to induce stratification as well as necessary for it, BSA- or FGF10-soaked beads were applied to frontal slices of E11.5 mandibles (Fig. S3C), close to the single-layered tongue epithelium. After 24 h, FGF induced a prominent thickening of the epithelium (Fig. 4F,G) composed of multilayered epithelial cells (Fig. 4F). Therefore, FGF is not only necessary but also sufficient for inducing epithelial stratification.

### Placode invagination depends on and is promoted by Shh signalling

Another pathway that shows regional expression and importance in early tooth germ is the Shh pathway (Cho et al., 2011; Dassule et al., 2000; Keränen et al., 1999). Genetic knockout using a keratin 14-driven promoter, active from ∼E11.75, has shown that Shh is required for tooth morphology but not cell differentiation (Dassule et al., 2000). Other studies suggested that, early in odontogenesis, Shh is involved in tooth initiation (Cobourne et al., 2001) and patterning (Cho et al., 2011). However, a fine temporal examination has not been performed to fit the varied roles of Shh to the separable early morphogenetic events we showed above. As with FGF, we exploited the temporal control of inhibition of Shh signalling using cyclopamine, which binds and antagonises the Shh transducer smoothened to effectively inhibit downstream targets (Fig. S3B, Fig. S4).

**Fig. 5.**
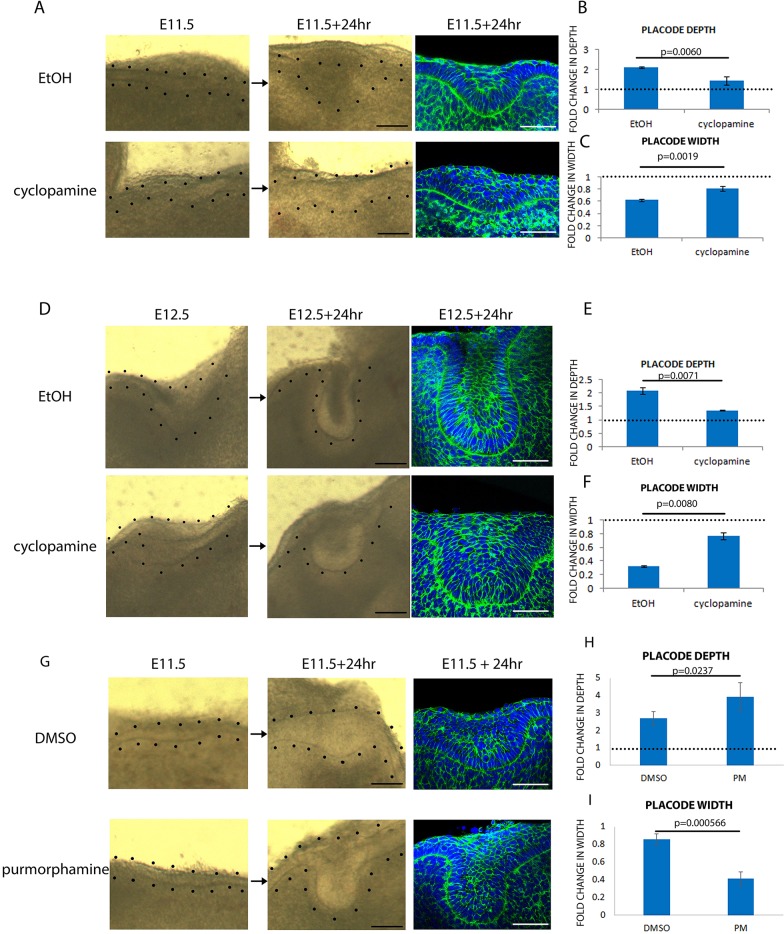
**Shh promotes epithelial bending in the tooth.** (A,D) Frontal slices of tooth placode cultured from E11.5 (A) or E12.5 (D) for 24 h with vehicle (ethanol) or smoothened inhibitor (cyclopamine). Left and middle columns are brightfield images showing the starting and ending morphology of the placodes; the right-hand column shows fixed placodes stained with DAPI (blue) and phalloidin (green). Black dots outline the epithelium. (B,C) Quantification of placode depth (B) and width (C) in A. (E,F) Quantification of placode depth (E) and width (F) in D. (G) Frontal slices of tooth placode cultured from E11.5 for 24 h with vehicle (DMSO) or smoothened activator (purmorphamine). Brightfield images and staining as in A,D. (H,I) Quantifications of placode depth (H) and width (I) in G. For all quantifications, *n*=9 specimens (three from each of three litters). Error bars indicate s.d. Scale bars: 50 μm.

In contrast to the effect of FGF inhibition, the inhibition of Shh signalling from E11.5 did not prevent stratification (Fig. 5A). However, cyclopamine treatment from E11.5 or E12.5 showed that although tooth placodes had stratified, the stratified structures were significantly shallower and wider than in control placodes (Fig. 5A-F).

To complement this loss-of-function result, we applied the Shh agonist purmorphamine (PM) (Wu et al., 2002) to E11.5 tooth, which slightly hyperactivates already active Shh responses in this tissue (Fig. S3C). Explant treatment resulted in the precocious formation of narrowed tooth bud necks at E11.5 plus 24 h, when DMSO controls still had a placodal morphology (Fig. 5G). Quantification confirmed that PM-treated teeth had significantly deeper buds and narrower necks than controls (Fig. 5H,I). None of the Shh signal-perturbing treatments altered placode volumes (Fig. S3G-I). We were unable to obtain a hyperactivation response to PM-soaked beads applied to tongue epithelium with or without added FGF (data not shown). Acceleration of invagination by PM tends to argue against the inhibition effects of cyclopamine being the result of non-specific cytotoxicity, but as a further control we stained cultured tissue with Lysotracker Red, which indicates cells dying through apoptosis or necrosis, and found no detectable elevation of staining in cyclopamine-treated explants at the concentration (20 µM) we used (Fig. S5).

Therefore, although both FGFs and Shh are expressed in early tooth germs, Shh signalling has a different role to FGFs: whereas FGF affects stratification but not invagination as such, Shh regulates invagination and thus the shape of the tooth bud but is dispensable for stratification.

### FGF but not Shh promotes proliferation in early tooth development

**Fig. 6.**
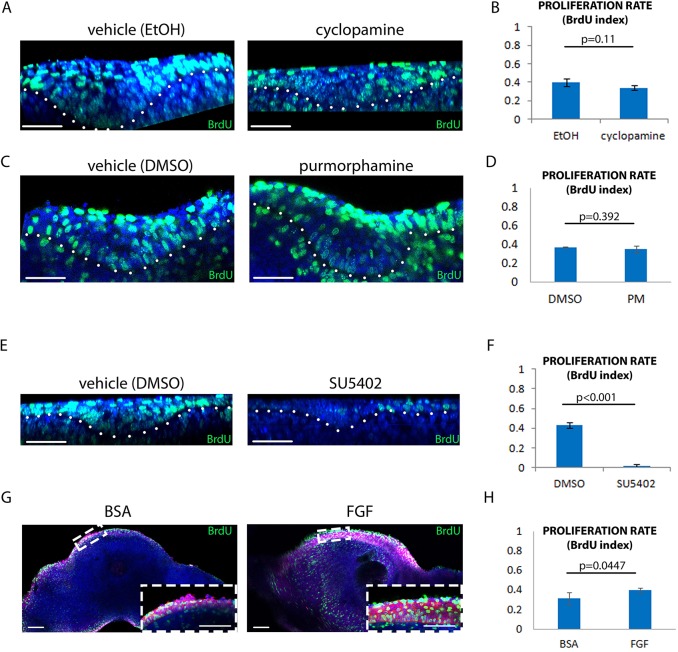
**FGF but not Shh promotes cell proliferation in the epithelium.** (A,C,E) Frontal view of E11.5 tooth placodes cultured for 24 h with vehicle (A, ethanol; C,E, DMSO), cyclopamine (A), purmorphamine (C) or SU5402 (E). Blue, DAPI; green, BrdU. White dots outline the epithelium. (B,D,F) BrdU index of A,C,E, respectively. *n*=3 each. (G) Frontal slices of mandibles applied with BSA-soaked or FGF-soaked beads. Blue, DAPI; green, BrdU; magenta, β-catenin (epithelium). Insets are magnified views of the tongue epithelium. (H) BrdU index of G. *n*=9 specimens (three from each of three litters). Error bars indicate s.d. Scale bars: 50 μm.

The phenotypes resulting from Shh signalling perturbation could be the result of effects on cell rearrangement or on cell proliferation or both. Indeed, it has been suggested that Shh does regulate cell proliferation in tooth development (Cobourne et al., 2001). To investigate these alternatives, we measured proliferation by BrdU labelling in cyclopamine-, PM- or Shh-treated cultures. None of these treatments, when applied to E11.5 explants for 24 h, had a significant effect on BrdU incorporation (Fig. 6A-D, Fig. S6), despite clear upregulation of transcriptional targets (Fig. S3, Fig. S6A). This is an important negative result because it implies that the Shh-dependent tissue shape changes observed in the developing tooth placode and bud are not proliferation dependent and, in the absence of significant cell death (data not shown), indicates that they involve cell rearrangement. The discrepancy with the findings of Cobourne et al. (2001) is discussed below. The lack of any effect on cell proliferation provides further evidence against the effects of the inhibitor resulting from non-specific toxicity, since dying cells do not divide.

We obtained a contrasting result with perturbations of FGF signalling. The similarity of the phenotypes induced by inhibiting proliferation or inhibiting FGF suggested that FGF could be acting via stimulation of cell proliferation. Indeed, when placodes were treated with SU to inhibit FGF signalling, proliferation was dramatically reduced (Fig. 6E,F), and when FGF-soaked beads were applied to non-placodal epithelium, stratification was induced and the proliferation rate showed a slight but statistically significant increase (Fig. 6G,H).

These data confirmed that whereas Shh is not essential for maintaining proliferation within the placode, FGF is both required and promotes cell proliferation.

### Shh regulates placode cell behaviours

**Fig. 7.**
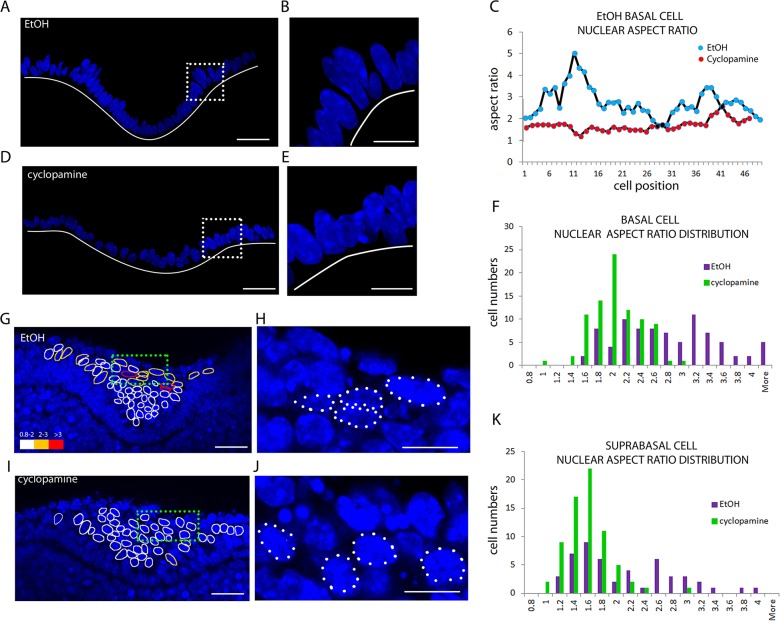
**Shh regulates basal and suprabasal cell elongation in the tooth.** (A,B,D,E) Confocal images of DAPI-stained nuclei in developing tooth germ explants with suprabasal and mesenchymal layers digitally removed to highlight basal layer nuclei. Solid white line indicates position of the basal lamina. The boxed regions in A,D are shown at higher magnification in B,E, respectively, highlighting the elongation of shoulder nuclei in controls (A,B) versus cyclopamine-treated (D,E) explants. (C) Nuclear aspect ratio plots across a control and treated placode showing change in the aspect ratio at shoulders. (F) Frequency distribution with respect to the basal cell nuclear aspect ratio in control and treated explants. (G-J) Confocal images of DAPI-stained nuclei in control (G,H) or cyclopamine-treated (I,J) explants. The boxed regions in G,I are magnified in H,J, respectively. Suprabasal nuclei are outlined to indicate aspect ratio (white=1; yellow=2-3; red ≥3). (K) Frequency distribution with respect to suprabasal cell nuclear aspect ratio. Data are representative of three independent experiments. Scale bars: 25 μm in A,D,G,I; 10 μm in B,E,H,J.

When we stained the slice cultures with phalloidin and DAPI, one apparent difference on the cellular level in Shh-perturbed cyclopamine-treated placodes was that suprabasal nuclei appeared somewhat rounder than those in control placodes (Fig. 7H,J). Changes in cell shape occur in morphogenetic processes when cells are highly motile or under tension, and the nucleus is a stiff structure that only deforms when the cell undergoes dramatic shape change under considerable tension (Friedl et al., 2011; Kettunen et al., 2007). To establish whether Shh might be regulating cell tension and/or motility in this system, we measured and mapped nuclear aspect ratios in control and cyclopamine-treated explants systematically. E11.5 frontal slices of tooth placodes were cultured for 1 day with or without inhibitor, fixed and stained with DAPI to reveal the nuclei (Fig. 7). Basal layer nuclei, both within and outside the placode, were consistently more elongated (as expected in a columnar epithelium) than suprabasal tissue nuclei, and so the layers were considered separately. In the basal layer of control tooth epithelium, most cells were columnar, with an average nuclear aspect ratio of 2 but with a striking distribution: in the ‘shoulder’ regions of the invagination, which are fated to eventually form the bud neck, nuclei were especially highly elongated (Fig. 7A,B), reaching aspect ratios greater than 3 (Fig. 7C). Cyclopamine treatment reduced basal nuclear aspect ratios, especially in the shoulders (Fig. 7C-E). The population frequency distribution of basal layer nuclear aspect ratios confirmed that when Shh signalling was downregulated, a population of elongated cell nuclei was lost (Fig. 7F). A similar effect was observed in suprabasal cells: inspection of control placodes revealed mostly rounded nuclei, but including a few more-elongated nuclei near the surface of the epithelium (Fig. 7G,H) with aspect ratios above 2.5, and these were apparent in a thick tail of the population distribution (Fig. 7K). (Note that superficial cells, which form the highly squamous peridermal layer, were excluded.) Cyclopamine-treated placodes lacked these elongated nuclei (Fig. 7I-K).

While nuclear shapes reflect applied forces, cell shapes as such can indicate motile behaviour. To begin to characterise cell shapes, mT/mG tissues with mosaically labelled cells were used. As with nuclei, cell elongation in both basal and suprabasal cell populations was impaired with cyclopamine treatment (Fig. S7). These results point to a potentially important subpopulation of cells in both the basal and suprabasal layer that are under Shh-dependent deformation. These cells and their deformation are likely to be crucial for morphogene­tic processes in tooth placode invagination.

## DISCUSSION

This study provides novel mechanistic insight into the cellular mechanisms that drive the formation of the molar tooth bud and, by homology, probably the formation of all ectodermal organ primordia. We distil two essential modules of morphogenetic cell behaviours, namely stratification and cell convergence, and identify a link to two major and well-established signalling pathways regulating tooth development, namely FGF and Shh signalling, respectively.

**Fig. 8.**
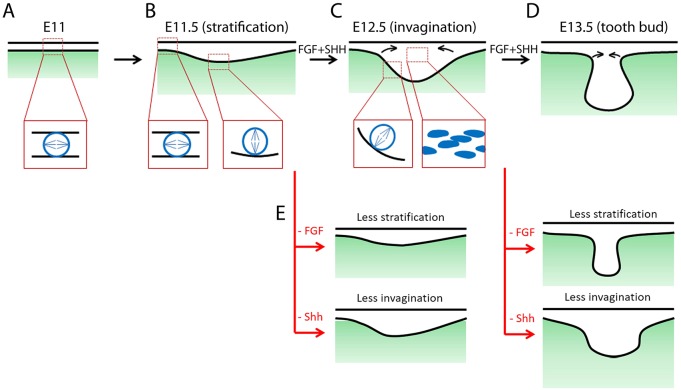
**Summary of mouse early tooth development.** (A) Flat oral epithelium before the formation of tooth placodes. Box illustrates parallel cell divisions in the monolayer. (B) Stratification of the tooth placode, showing parallel divisions in a non-placodal region and perpendicular divisions in the placode. (C) Placode invagination and constriction of the neck. Left box shows perpendicular division in basal placodal cells. Right box illustrates elongated suprabasal cells constricting the neck. (D) Bud stage tooth. (E) Inhibition of FGF or Shh from E11.5 or E12.5 inhibits stratification or invagination, respectively.

Our study does not address whether the effects of applied factors and inhibitors act directly on epithelium or somehow via mesenchymal signalling. In the case of Shh, at least, the apparently normal development of mesenchyme in an epithelium-specific knockout of the receptor smoothened suggests that the signalling acts primarily on the epithelium itself (Gritli-Linde et al., 2002). However, the use of inhibitors side-steps some of the complexities of multiple forms of the ligands (particularly FGFs) and the times and locations of gene expression that have been the focus of the field in recent years. It also addresses the uncertainties about phenotypes observed in tissue-specific knockouts driven by promoters such as keratin 14, which is only expressed after the appearance of the earliest placode (Cho et al., 2011; Dassule et al., 2000) and cannot prevent signalling perduring beyond the onset of Cre expression. FGF signalling-dependent perpendicular cell divisions act first to generate suprabasal cells (stratification). Then, Shh signalling acts on these cells to deform and rearrange them into a deep placode and ultimately an invaginated bud (Fig. 8). Although FGF has been linked to proliferation in tooth development (Ejeian et al., 2014), our findings go beyond a general examination of an ‘arrested’ tooth by demarcating the roles of FGFs in early odontogenesis as being proliferation only and that of Shh as being cell rearrangement only. Many other signals are known to be important in tooth formation, but our observations provide a new way to rationalise a body of genetics and developmental literature on ectodermal organ phenotypes and provide a framework for linking molecular mechanisms to tissue-level structures via the actions of cells. With this framework, we could start to investigate the complicated epithelium-mesenchyme interaction and genetic network at cellular resolution.

Of course, although these processes of stratification and invagination are experimentally separable, they are also intimately linked. Without stratification, we showed that later invagination cannot occur. Likewise, during invagination cells must migrate centripetally and intercalate to form a stack, which has the effect of concentrating the stratification at the centre of the placode. Thus, in the molar tooth, just as in the hair follicle, centripetal cell movement is a significant part of placode formation (Ahtiainen et al., 2014). However, this study goes beyond what is known in the hair follicle: our findings reveal the mechanism of generation of the converging cells, that converging cells are suprabasal, and that suprabasal cells very likely converge towards the centre of the molar tooth placode. These processes drive not only placode thickening but also subsequent epithelial bending, placode narrowing and bud neck formation. We have also shown that this implicated cell rearrangement requires, and it is driven by, Shh signalling.

One very influential study (Osman and Ruch, 1975) suggested that in the mouse ‘dental lamina’, vertical spindles are associated with epithelial thickening. The ‘dental lamina’ in mouse, now referred to as the ‘odontogenic band’ (Fraser et al., 2004), includes the diastema, which does not produce teeth. The diastema undergoes modest stratification (e.g. two to three cell diameters) around the time of tooth formation (Yamamoto et al., 2005), and so it is unclear whether Osman and Ruch's observations were tooth field-specific or not. Our studies have shown by virtue of higher resolution – both spatial and temporal – that vertical spindles are a property of much, or all, of the odontogenic band, but begin earlier in the region of the prospective molar than elsewhere. Thus, our findings suggest that vertical spindle orientation causes at least some molar placode-specific stratification, despite it not being a unique behaviour of the molar placode.

Our finding that, without proliferation, the dental placode was not able to stratify at all not only demonstrated that (predominantly perpendicular) cell divisions are necessary for all detectable suprabasal layer formation, but also ruled out the possibility that, independently of proliferation, simple delamination of cells from the basal layer to the suprabasal layer is sufficient for stratification. However, this finding does not rule out more complex extrusion scenarios in which planar cell division triggers the extrusion of one of the daughter cells (Kulukian and Fuchs, 2013).

FGF signalling, although capable of regulating cell motility during morphogenesis in some contexts (Li et al., 2014), is unlikely to be affecting cell motility in the molar placode since, following FGF inhibition, cell rearrangements still occur essentially normally and cell shapes are unaffected.

On the other hand, Shh signalling is required for neither cell proliferation nor growth in size during early tooth development, but complements the role of FGF by determining the shape of the tooth placode. The shallower and wider tooth that we obtained from perturbing Shh with chemical inhibitors at different developmental stages was similar to that of the *Shh* mutants (Dassule et al., 2000). The latter was previously interpreted as a defect in proliferation (Cobourne et al., 2001), which was different from what we found upon thorough 3D examination of the entire tooth placode. Therefore, the contrast with the findings of the previous report might be due to inadequate sampling of tissue sections that only included a subset of the placodal cell populations. We can now reinterpret this phenotype as primarily a defect in convergent cell migration and intercalation of the type reported in hair placodes (Ahtiainen et al., 2014), as suggested by the change of cell shape, which normally implies directional cell migration or tension. Moreover, because a role of Shh in chemotaxis has been discovered (Bijlsma et al., 2012), it is plausible that the regional expression of Shh at the bottom of the molar placode acts as a chemoattractant for the convergence of suprabasal cells. However, we cannot rule out the possibility that Shh regulates the specification or polarisation of motile cells (rather than motility as such), especially since perturbations of Shh signalling do alter the size of the *Shh* expression domain (data not shown).

Finally, these findings have raised several further questions in the development of ectodermal organs. For example, how is the spindle orientation regulated? What are the movements and forces that cells undergo to create the shape of the bud (or peg or duct, in the case of other ectodermal organs)? To what extent are these mechanisms actually conserved across the homologous ectodermal organs? Does Shh directly trigger cell motility or does it act via, for example, Wnt signalling (or vice versa), as proposed for hair placodes (Ahtiainen et al., 2014)? The general morphogenetic mechanism by which ectodermal organs are initiated lies in the answers to these questions.

## MATERIALS AND METHODS

### Mouse strains and staging of embryos

All work with animals was compliant with UK Home Office regulations and King's College London Ethics Committee approval. Mosaic cell labelling was achieved by injecting 1 mg tamoxifen (Sigma) from a 10 mg/ml stock in autoclaved corn oil and 2 mg progesterone (Sigma) from a 10 mg/ml stock into a pregnant female mT/mG mouse [Gt(ROSA)26Sortm4(ACTB-tdTomato,-EGFP) Luo/J; Jackson Laboratories strain 007576] crossed with a tamoxifen-inducible Cre male B6.Cg-Tg(CAG-cre/Esr1*)5Amc/J (Jackson Laboratories strain 004682). Injection was performed at E9.5 and embryos were harvested at E11.5. mT/mG mice and CD-1 mice were sacrificed by cervical dislocation. Embryos were staged by nominal age (noon of the day of vaginal plug detection taken as E0.5) and the number of somites.

### Explant culture and treatments

Slice culture of tooth placodes was as described (Alfaqeeh and Tucker, 2013). Briefly, embryo heads dissected in Advanced Dulbecco's Modified Eagle Medium F12 (DMEM F12) were bisected, chopped into 250 μm frontal slices on a McIlwain Tissue Chopper and cultured on a transparent filter (pore size 0.4 μm, Corning #353090) supported by a steel mesh (Goodfellow, FE228710) in DMEM F12 with 1% penicillin-streptomycin (Sigma) at 37°C and 5% CO_2_. Whole mandibles were prepared as previously described (Hardcastle et al., 1998) and cultured in the same way.

For proliferation inhibition, explants were cultured with 5 mM hydroxyurea (Sigma) and 5 μg/ml aphidicolin (Santa Cruz Biotechnology) for 24 h before harvest. FGF signalling inhibitor SU5402 (Merck) was diluted from 10 mM DMSO stock to 20 μM. Shh signalling inhibitor cyclopamine (Sigma) was diluted from 10 mg/ml ethanol stock to 20 μM. Shh agonist purmorphamine (Calbiochem) was used at 200 μM, diluted from a 50 mM DMSO stock. Shh protein (R&D Systems) was used at 1.5 μg/ml, diluted from a 100 μg/ml stock. For BrdU labelling, mandibular explants were cultured as above with 10 μM BrdU for 1 h then submerged in medium for 1 h before fixation. Lysotracker Red (ThermoFisher) was used at 5 μM in culture medium to stain for dead and dying cells as described (Fogel et al., 2012).

For bead experiments, Affi-Gel Blue beads (Bio-Rad, 153-7302) were dried then soaked in 0.5% BSA in PBS at 4°C overnight (o/n) before use as control. Heparin beads (Sigma, 100-200 mesh) were prepared in 100 μg/ml FGF10 protein (R&D Systems, 6224-FG-02S) at 4°C o/n before use. Frontal slices of mandibles dissected from E11.5 CD-1 mice were cultured as above with beads applied close to the tongue epithelium.

All experiments were repeated for at least nine experimental and nine littermate control specimens, i.e. at least three embryos for each condition from three separate litters.

### *In situ* hybridisation

Digoxigenin-labelled whole-mount *in situ* hybridisation was performed according to Economou et al. (2012). Probes used were: *Shh* (Economou et al., 2012), *Ptc1* (Cobourne et al., 2004) and *Pea3* (*Etv4*) (Gardiner et al., 2012).

### Immunostaining and imaging

Specimens were fixed in MEMFA (0.1 M MOPS pH 7.4, 2 mM EGTA, 1 mM MgSO_4_, 3.7% formaldehyde) for 4 h at room temperature (RT) or 4°C o/n, except for E-cadherin staining for which specimens were fixed with precooled Dent's fix (80% methanol, 20% DMSO) at −20°C o/n. For BrdU staining, specimens were post-treated with 4 M HCl for 15 min at RT. Specimens were washed three times with PBS containing 0.1% Triton X-100 and blocked with 10% goat serum (Sigma) for 1 h at RT. Antibody dilutions and chemical stains were in block at the following dilutions: anti-β-catenin 1:500 (Sigma, C2206), anti-γ-tubulin 1:400 (Sigma, T5192), anti-E-cadherin 1:2000 (DECMA-1, Sigma, U3254), anti-laminin 1:500 (Sigma, L9393), anti-BrdU 1:200 (Becton Dickinson, 347580), Alexa 488/568 anti-mouse/rabbit/rat 1:500 (Life Technologies, A21208, A11008, A10042 and A10037), fluorescent phalloidin (Life Technologies) 1:200, and DAPI (Life Technologies) 1:10,000. Incubations were at 4°C o/n. For whole-mount specimens (>80 μm thick) post-fixation (MEMFA, RT, 1 h) was performed after immunostaining then Scale A2 and Scale B4 solutions (Hama et al., 2011) were used to clear tissues for confocal imaging.

Brightfield images of cultured slices were taken with a Nikon SMZ-U stereomicroscope and a MicroPublisher 5.0 RTV camera with transilluminated base. Fluorescent images were captured on a Leica SP5 confocal microscope with an HCX PL APO CS 40× oil (N.A. 1.25) or an HCX PL APO 63×/1.3 GLYC CORR CS (21°C) objective.

### Image analyses

To measure spindle orientation, whole-mount mandible specimens stained with DAPI or for β-catenin and γ-tubulin were imaged *en face* from the surface of the oral epithelium to the basal lamina. Using Fiji/ImageJ (Schindelin et al., 2012), acquired confocal stacks were resliced frontally and a line in the frontal plane was drawn between centrosomes located inside each mitotic cell (scrolling between image slices when necessary). The acute angle between that line and its projection on the adjacent basal lamina was measured with the Fiji angle tool. Angles between 0° and 30° were categorised as parallel to the basal lamina, 30°-60° as oblique and 60°-90° as perpendicular. Six mandibles from two litters were analysed for each stage.

In slice cultures, placode depth was defined as the distance from the surface of the epithelium to the deepest part of the invagination, and the width as the distance between the most convex surface points on the lingual and labial sides of the invagination.

For 3D reconstruction of the suprabasal tissue volume or whole placode volume, whole-mount tooth placodes, stained for E-cadherin or laminin, respectively, were imaged *en face* to obtain confocal stacks. The punctate E-cadherin (DECMA-1) staining was smoothed to enable 3D rendering, first by replacing each slice with a *z*-projection of 50 adjacent slices (25 above, 25 below) and then applying a Fiji smoothing filter. Laminin staining required only the smoothing filter. 3D rendering was generated using 3D viewer in Fiji. To measure the invaginated volume of the placodes, frontal slices of *en face* laminin-stained confocal stacks were manually cropped at a line level with the underside of uninvaginated epithelium to leave only the area of invaginated tissue. This area was captured semi-automatically (‘Magic Wand' command) and the volume calculated as the area multiplied by the *z*-step between slices and then summed for all the slices.

BrdU labelling was quantified manually by counting the proportion of DAPI-stained nuclei that were also positive for BrdU in three different image slices (at least 10 μm apart) chosen from frontally resliced *en face* confocal image stacks of each placode.

Nuclear aspect ratios were defined as the maximum length of the longest axis observable in frontal sections of a nucleus divided by its width at the long-axis mid-point. For this measurement, thick (250 μm) tissue slices were imaged using optimal *z* resolution confocal scan, and the long axis was determined by *z* projections that included entire cells. For mosaically labelled cells, the (*x*, *y*, *z*) coordinates of medial, lateral, top and bottom ends were recorded, and the lengths of the major and minor axes of cells were calculated as the distances between medial-lateral and top-bottom pairs, respectively. Aspect ratios were defined as the ratio between the lengths of major and minor axes.

### Statistics

All *P*-values were calculated using a Student's two-tailed *t*-test in Microsoft Excel.

## References

[DEV130187C1] Ahtiainen L., Lefebvre S., Lindfors P. H., Renvoisé E., Shirokova V., Vartiainen M. K., Thesleff I. and Mikkola M. L. (2014). Directional cell migration, but not proliferation, drives hair placode morphogenesis. *Dev. Cell* 28, 588-602. 10.1016/j.devcel.2014.02.003 24636260

[DEV130187C2] Alfaqeeh S. A. and Tucker A. S. (2013). The slice culture method for following development of tooth germs in explant culture. *J. Vis. Exp.*, e50824 10.3791/50824 24300332 PMC3990833

[DEV130187C3] Balinsky B. I. (1950). On the prenatal growth of the mammary gland rudiment in the mouse. *J. Anat.* 84, 227-235. 15436328 PMC1273299

[DEV130187C4] Biggs L. C. and Mikkola M. L. (2014). Early inductive events in ectodermal appendage morphogenesis. *Semin. Cell Dev. Biol.* 25-26, 11-21. 10.1016/j.semcdb.2014.01.007 24487243

[DEV130187C5] Bijlsma M. F., Damhofer H. and Roelink H. (2012). Hedgehog-stimulated chemotaxis is mediated by smoothened located outside the primary cilium. *Sci. Signal.* 5, ra60 10.1126/scisignal.2002798 22912493 PMC4557959

[DEV130187C6] Chiu M. L., Jones J. C. and O'Keefe E. J. (1992). Restricted tissue distribution of a 37-kD possible adherens junction protein. *J. Cell Biol.* 119, 1689-1700. 10.1083/jcb.119.6.1689 1469056 PMC2289738

[DEV130187C7] Cho S.-W., Kwak S., Woolley T. E., Lee M.-J., Kim E.-J., Baker R. E., Kim H.-J., Shin J.-S., Tickle C., Maini P. K. et al. (2011). Interactions between Shh, Sostdc1 and Wnt signaling and a new feedback loop for spatial patterning of the teeth. *Development* 138, 1807-1816. 10.1242/dev.056051 21447550

[DEV130187C8] Cobourne M. T., Hardcastle Z. and Sharpe P. T. (2001). Sonic hedgehog regulates epithelial proliferation and cell survival in the developing tooth germ. *J. Dent. Res.* 80, 1974-1979. 10.1177/00220345010800110501 11759005

[DEV130187C9] Cobourne M. T., Miletich I. and Sharpe P. T. (2004). Restriction of sonic hedgehog signalling during early tooth development. *Development* 131, 2875-2885. 10.1242/dev.01163 15151988

[DEV130187C10] da Silva S. M. and Vincent J.-P. (2007). Oriented cell divisions in the extending germband of Drosophila. *Development* 134, 3049-3054. 10.1242/dev.004911 17652351

[DEV130187C11] Dassule H. R., Lewis P., Bei M., Maas R. and McMahon A. P. (2000). Sonic hedgehog regulates growth and morphogenesis of the tooth. *Development* 127, 4775-4785. 11044393 10.1242/dev.127.22.4775

[DEV130187C12] Economou A. D., Ohazama A., Porntaveetus T., Sharpe P. T., Kondo S., Basson M. A., Gritli-Linde A., Cobourne M. T. and Green J. B. A. (2012). Periodic stripe formation by a Turing mechanism operating at growth zones in the mammalian palate. *Nat. Genet.* 44, 348-351. 10.1038/ng.1090 22344222 PMC3303118

[DEV130187C13] Ejeian F., Baharvand H. and Nasr-Esfahani M. H. (2014). Hedgehog signalling is dispensable in the proliferation of stem cells from human exfoliated deciduous teeth. *Cell Biol. Int.* 38, 480-487. 10.1002/cbin.10227 24353013

[DEV130187C14] Fogel J. L., Thein T. Z. T. and Mariani F. V. (2012). Use of LysoTracker to detect programmed cell death in embryos and differentiating embryonic stem cells. *J. Vis. Exp.*, e4254 10.3791/4254 PMC3490301 23092960

[DEV130187C15] Fraser G. J., Graham A. and Smith M. M. (2004). Conserved deployment of genes during odontogenesis across osteichthyans. *Proc. R. Soc. B Biol. Sci.* 271, 2311-2317. 10.1098/rspb.2004.2878 PMC1691870 15556883

[DEV130187C16] Friedl P., Wolf K. and Lammerding J. (2011). Nuclear mechanics during cell migration. *Curr. Opin. Cell Biol.* 23, 55-64. 10.1016/j.ceb.2010.10.015 21109415 PMC3073574

[DEV130187C17] Gardiner J. R., Jackson A. L., Gordon J., Lickert H., Manley N. R. and Basson M. A. (2012). Localised inhibition of FGF signalling in the third pharyngeal pouch is required for normal thymus and parathyroid organogenesis. *Development* 139, 3456-3466. 10.1242/dev.079400 22912418 PMC3424047

[DEV130187C18] Gritli-Linde A., Bei M., Maas R., Zhang X. M., Linde A. and McMahon A. P. (2002). Shh signaling within the dental epithelium is necessary for cell proliferation, growth and polarization. *Development* 129, 5323-5337. 10.1242/dev.00100 12403705

[DEV130187C19] Hama H., Kurokawa H., Kawano H., Ando R., Shimogori T., Noda H., Fukami K., Sakaue-Sawano A. and Miyawaki A. (2011). Scale: a chemical approach for fluorescence imaging and reconstruction of transparent mouse brain. *Nat. Neurosci.* 14, 1481-1488. 10.1038/nn.2928 21878933

[DEV130187C20] Hardcastle Z., Mo R., Hui C. C. and Sharpe P. T. (1998). The Shh signalling pathway in tooth development: defects in Gli2 and Gli3 mutants. *Development* 125, 2803-2811. 9655803 10.1242/dev.125.15.2803

[DEV130187C21] Harris W. A. and Hartenstein V. (1991). Neuronal determination without cell division in Xenopus embryos. *Neuron* 6, 499-515. 10.1016/0896-6273(91)90053-3 1901716

[DEV130187C22] Hosokawa R., Deng X., Takamori K., Xu X., Urata M., Bringas P. Jr and Chai Y. (2009). Epithelial-specific requirement of FGFR2 signaling during tooth and palate development. *J. Exp. Zoolog. B Mol. Dev. Evol.* 312B, 343-350. 10.1002/jez.b.21274 PMC2896559 19235875

[DEV130187C23] Jernvall J. and Thesleff I. (2000). Reiterative signaling and patterning during mammalian tooth morphogenesis. *Mech. Dev.* 92, 19-29. 10.1016/S0925-4773(99)00322-6 10704885

[DEV130187C24] Keränen S. V., Kettunen P., Aberg T., Thesleff I. and Jernvall J. (1999). Gene expression patterns associated with suppression of odontogenesis in mouse and vole diastema regions. *Dev. Genes Evol.* 209, 495-506. 10.1007/s004270050282 10415326

[DEV130187C25] Kettunen P., Spencer-Dene B., Furmanek T., Kvinnsland I. H., Dickson C., Thesleff I. and Luukko K. (2007). Fgfr2b mediated epithelial–mesenchymal interactions coordinate tooth morphogenesis and dental trigeminal axon patterning. *Mech. Dev.* 124, 868-883. 10.1016/j.mod.2007.09.003 17951031

[DEV130187C26] Kulukian A. and Fuchs E. (2013). Spindle orientation and epidermal morphogenesis. *Philos. Trans. R. Soc. Lond. B Biol. Sci.* 368, 20130016 10.1098/rstb.2013.0016 24062586 PMC3785966

[DEV130187C27] Lechler T. and Fuchs E. (2005). Asymmetric cell divisions promote stratification and differentiation of mammalian skin. *Nature* 437, 275-280. 10.1038/nature03922 16094321 PMC1399371

[DEV130187C28] Li C.-Y., Prochazka J., Goodwin A. F. and Klein O. D. (2014). Fibroblast growth factor signaling in mammalian tooth development. *Odontology* 102, 1-13. 10.1007/s10266-013-0142-1 24343791 PMC9202745

[DEV130187C29] Linde A. (1984). *Dentin and Dentinogenesis*. Boca Raton: CRC Press.

[DEV130187C30] Magerl M., Tobin D. J., Müller-Röver S., Hagen E., Lindner G., McKay I. A. and Paus R. (2001). Patterns of proliferation and apoptosis during murine hair follicle morphogenesis. *J. Invest. Dermatol.* 116, 947-955. 10.1046/j.0022-202x.2001.01368.x 11407986

[DEV130187C31] Mailleux A. A., Spencer-Dene B., Dillon C., Ndiaye D., Savona-Baron C., Itoh N., Kato S., Dickson C., Thiery J. P. and Bellusci S. (2002). Role of FGF10/FGFR2b signaling during mammary gland development in the mouse embryo. *Development* 129, 53-60. 11782400 10.1242/dev.129.1.53

[DEV130187C32] Mikkola M. L. and Millar S. E. (2006). The mammary bud as a skin appendage: unique and shared aspects of development. *J. Mammary Gland Biol. Neoplasia* 11, 187-203. 10.1007/s10911-006-9029-x 17111222

[DEV130187C33] Montell D. J. (2008). Morphogenetic cell movements: diversity from modular mechanical properties. *Science* 322, 1502-1505. 10.1126/science.1164073 19056976

[DEV130187C34] Nanci A. and Ten Cate A. R. (2013). *Ten Cate's Oral Histology: Development, Structure, and Function*, 8th edn St. Louis: Elsevier.

[DEV130187C35] Neubüser A., Peters H., Balling R. and Martin G. R. (1997). Antagonistic interactions between FGF and BMP signaling pathways: a mechanism for positioning the sites of tooth formation. *Cell* 90, 247-255. 10.1016/S0092-8674(00)80333-5 9244299

[DEV130187C36] Ohuchi H., Hori Y., Yamasaki M., Harada H., Sekine K., Kato S. and Itoh N. (2000). FGF10 acts as a major ligand for FGF receptor 2 IIIb in mouse multi-organ development. *Biochem. Biophys. Res. Commun.* 277, 643-649. 10.1006/bbrc.2000.3721 11062007

[DEV130187C37] Osman A. and Ruch J. V. (1975). [Topographical pattern of mitosis in the odontogenic regions of the mandible in the mouse embryo]. *J. Biol. Buccale* 3, 117-132. 1058184

[DEV130187C38] Panousopoulou E. and Green J. B. A. (2014). Spindle orientation processes in epithelial growth and organisation. *Semin. Cell Dev. Biol.* 34, 124-132. 10.1016/j.semcdb.2014.06.013 24997348

[DEV130187C39] Park B. Y. and Saint-Jeannet J. P. (2010). *Induction and Segregation of the Vertebrate Cranial Placodes*. San Rafael: Morgan & Claypool Life Sciences. 21452441

[DEV130187C40] Petiot A., Conti F. J. A., Grose R., Revest J.-M., Hodivala-Dilke K. M. and Dickson C. (2003). A crucial role for Fgfr2-IIIb signalling in epidermal development and hair follicle patterning. *Development* 130, 5493-5501. 10.1242/dev.00788 14530295

[DEV130187C41] Pispa J. and Thesleff I. (2003). Mechanisms of ectodermal organogenesis. *Dev. Biol.* 262, 195-205. 10.1016/S0012-1606(03)00325-7 14550785

[DEV130187C42] Prochazka J., Pantalacci S., Churava S., Rothova M., Lambert A., Lesot H., Klein O., Peterka M., Laudet V. and Peterkova R. (2010). Patterning by heritage in mouse molar row development. *Proc. Natl. Acad. Sci. USA* 107, 15497-15502. 10.1073/pnas.1002784107 20709958 PMC2932592

[DEV130187C43] Propper A. Y. (1978). Wandering epithelial cells in the rabbit embryo milk line. A preliminary scanning electron microscope study. *Dev. Biol.* 67, 225-231. 10.1016/0012-1606(78)90311-1 720754

[DEV130187C44] Schindelin J., Arganda-Carreras I., Frise E., Kaynig V., Longair M., Pietzsch T., Preibisch S., Rueden C., Saalfeld S., Schmid B. et al. (2012). Fiji: an open-source platform for biological-image analysis. *Nat. Methods* 9, 676-682. 10.1038/nmeth.2019 22743772 PMC3855844

[DEV130187C45] Schmidt-Ullrich R., Tobin D. J., Lenhard D., Schneider P., Paus R. and Scheidereit C. (2006). NF-kappaB transmits Eda A1/EdaR signalling to activate Shh and cyclin D1 expression, and controls post-initiation hair placode down growth. *Development* 133, 1045-1057. 10.1242/dev.02278 16481354

[DEV130187C46] Sennett R. and Rendl M. (2012). Mesenchymal–epithelial interactions during hair follicle morphogenesis and cycling. *Semin. Cell Dev. Biol.* 23, 917-927. 10.1016/j.semcdb.2012.08.011 22960356 PMC3496047

[DEV130187C47] St Johnston D. and Sanson B. (2011). Epithelial polarity and morphogenesis. *Curr. Opin. Cell Biol.* 23, 540-546. 10.1016/j.ceb.2011.07.005 21807488

[DEV130187C48] Tabler J. M., Yamanaka H. and Green J. B. A. (2010). PAR-1 promotes primary neurogenesis and asymmetric cell divisions via control of spindle orientation. *Development* 137, 2501-2505. 10.1242/dev.049833 20573701

[DEV130187C49] Wessells N. K. and Roessner K. D. (1965). Nonproliferation in dermal condensations of mouse vibrissae and pelage hairs. *Dev. Biol.* 12, 419-433. 10.1016/0012-1606(65)90007-2 5884353

[DEV130187C50] Williams S. E., Ratliff L. A., Postiglione M. P., Knoblich J. A. and Fuchs E. (2014). Par3–mInsc and Galphai3 cooperate to promote oriented epidermal cell divisions through LGN. *Nat. Cell Biol.* 16, 758-769. 10.1038/ncb3001 25016959 PMC4159251

[DEV130187C51] Wodarz A. and Huttner W. B. (2003). Asymmetric cell division during neurogenesis in Drosophila and vertebrates. *Mech. Dev.* 120, 1297-1309. 10.1016/j.mod.2003.06.003 14623439

[DEV130187C52] Wu X., Ding S., Ding Q., Gray N. S. and Schultz P. G. (2002). A small molecule with osteogenesis-inducing activity in multipotent mesenchymal progenitor cells. *J. Am. Chem. Soc.* 124, 14520-14521. 10.1021/ja0283908 12465946

[DEV130187C53] Yamamoto H., Cho S.-W., Song S.-J., Hwang H.-J., Lee M.-J., Kim J.-Y. and Jung H.-S. (2005). Characteristic tissue interaction of the diastema region in mice. *Arch. Oral Biol.* 50, 189-198. 10.1016/j.archoralbio.2004.11.010 15812993

[DEV130187C54] Yuan G. H., Zhang L., Zhang Y. D., Fan M. W., Bian Z. and Chen Z. (2008). Mesenchyme is responsible for tooth suppression in the mouse lower diastema. *J. Dent. Res.* 87, 386-390. 10.1177/154405910808700412 18362325

